# Media Portrayals of Emerging and Older Adults during COVID-19: Perceptions of Bias Among College Students

**DOI:** 10.1093/geroni/igab046.2298

**Published:** 2021-12-17

**Authors:** Taylor Pestritto, Katherine King, Mikala Mikrut, Kirsten Graham

**Affiliations:** 1 William James, Newton, Massachusetts, United States; 2 William James College, William James College, Massachusetts, United States; 3 Southern Utah University, Cedar City, Utah, United States

## Abstract

This study explores media consumption and perceptions of media bias against both older adults and emerging adults during the COVID-19 pandemic. As part of a larger study, 99 students with a mean age of 20.54 (SD = 2.97) completed an online survey in early 2020. Individuals whose media consumption had increased were significantly more likely to report that young adults have been portrayed worse, and older adults better, since the start of COVID-19. Qualitative responses demonstrated broad awareness of ageist and adultist themes in media portrayals of both age groups, e.g., that young adults are careless and reckless whereas older adults are vulnerable and in need of protection. Results suggest that the media is perceived to be perpetuating age-related biases and may be enhancing intergenerational discord at a time when generational unity is needed.

